# Effectiveness of using a chatbot to promote adherence to home physiotherapy after total knee replacement, rationale and design of a randomized clinical trial

**DOI:** 10.1186/s12891-023-06607-3

**Published:** 2023-06-15

**Authors:** José-María Blasco, Beatriz Díaz-Díaz, Celedonia Igual-Camacho, José Pérez-Maletzki, David Hernández-Guilén, Sergio Roig-Casasús

**Affiliations:** 1 Group in Physiotherapy in the Ageing Processess: Social and Healthcare Strategies, Departament de Fisioteràpia, Universitat de València, Calle Gascó Oliag 5, Valencia, 46010 Spain; 2 IRIMED, Joint Research Unit La Fe-UV, Valencia, Spain; 3 Hospital Clínic i Universitari de València, Valencia, Spain; 4 Hospital Universitari i Politècnic la Fe de València, Valencia, Spain

**Keywords:** Total knee replacement, Adherence, compliance, Chatbot

## Abstract

**Background:**

Rehabilitation is essential to optimize outcomes after surgical procedures in musculoskeletal disorders. However, adherence to rehabilitation continues to be an important barrier, since compliance with the programs is not always as desired, which may have a negative impact on clinical results.

**Methods:**

Randomized controlled trial aimed at to determining the effectiveness of using a virtual assistant (i.e., chatbot) to promote adherence to home rehabilitation. Overall, seventy patients under 75, undergoing total knee replacement, who have a personal smartphone and are familiar with its use, will be assigned into the control (standard care) or the experimental (standard care plus virtual assistant) group. Adherence (primary outcome) will be assessed three months after surgery. The WOMAC questionnaire, knee pain and system usability scale will be also outcomes of interest at three months and one year. Overall, an analysis of variance will look for possible time, group and time*group interactions.

**Discussion:**

The expected result is to determine whether the use of a chatbot that interacts with the patient can increase adherence to post-surgical home physiotherapy, and result in better clinical results (functional and pain) than standard care.

**Trial registration:**

clinicaltrials.gov id. NCT05363137

**Supplementary Information:**

The online version contains supplementary material available at 10.1186/s12891-023-06607-3.

## Introduction

Musculoskeletal disorders (MD) are the leading cause of pain and disability [1]. Physiotherapy plays a fundamental role in the recovery, but low adherence, reported to be from 65% to as low as 20% [2–4], continue to be one of the main barriers [5], especially regarding in-home and non-face-to-face interventions [2, 6].

Understanding adherence as the degree to which a person’s behavior corresponds to the agreed recommendations of a health care provider (WHO, 2003), adherent patients may have better clinical outcome [7–9], whereas poor compliance has a negative influence, with likely consequences on socio-sanitary costs [10].

The current limitation of resources has caused healthcare systems to progressively implement non-face-to-face rehabilitation programs, based on education and information brochures [11, 12]. A situation enhanced by the recent health pandemic that has driven a paradigm shift, especially in cases of risk such as older adults, people with comorbidities, as well as people with mobility difficulties, living in isolated areas or with any other similar situation that makes health care difficult [13].

Current trends point to the use of information and communication technologies (ICT) and digital media as a solution to administer home treatments [14–17]. However, since non-compliance rates are one of the main barriers, the digital applications of physiotherapy should be aimed not only at improving clinical results, but also at fostering adherence. This will be achieved through interactive and accessible environments that promote self-efficacy and behavioral changes, setting measurable and achievable goals and reporting on progress.

### ICT, rehabilitation and adherence

The use of ICT in rehabilitation has evolved over time, since the 1980s when it was proposed to emphasize follow-up with phone calls [18], towards other solutions such as pre-recorded videos for use and interaction with the patient [19], interactive videoconferences [15], and technologies based on sensors and remote monitoring [20]. In addition, interventions based on virtual equipment and environments, specifically designed for rehabilitation or not, have been common, among which the interest in devices such as the Wii® have stood out [21–23].

In recent years, smartphones have revolutionized communication, with new opportunities also in the medical field. Especially considering factors like half of smartphone users use their devices for health information [24], or that a fifth part use applications related to this area [25]. Due to this, the range of health-oriented mobile applications (Apps) available for health professionals, patients, students and the general population is increasing [26].

Although adherence is a topic that is attracting more and more interest, the applications do not usually specifically aim to promote this aspect. In the field of healthcare there are a large number of mobile applications or electronic devices used for this purpose (e.g., the electronic pillbox) [27], but are mostly oriented to taking medications, helping the patient with reminders. Other applications do have additional features that promote adherence to medication without forgetting adherence to treatment. However, very little research has been done to assess their effectiveness for the purposes for which they were designed or the level of acceptance among users [28].

### Scientific proposal and objective

Smart mobile devices are possibly the technology that currently offers the greatest potential. Apps are the main trend today [28]. As an alternative, this project proposes the use of a virtual assistant, acting as a conversational healthcare provider by means of a chatbot. Chatbots can have different degrees of complexity; the most advanced ones, are designed with artificial intelligence, through natural language processing (NLP). The assistants are responsible for simulating a human conversation, which can be very useful for guiding the patient in the physiotherapy program. Chatbots, mainly used for commercial purposes, have been increasingly appraised in healthcare [29, 30]. However, their use to manage musculoskeletal disorders is still understudied.

Given the above factors, this research aims to determine the effectiveness of a virtual assistant chatbot, that communicates via an instant messaging service application, to promote compliance and adherence to home physiotherapy programs in people undergoing total knee replacement. The hypothesis is that the use of a chatbot that interacts with a patient undergoing total knee replacement through an instant messaging service will increase adherence to home physiotherapy, what improve clinical outcome (function and pain).

### Study population

More than one hundred MD have been classified (WHO, 2019). The scientific proposal will be transferable to the high number conditions that require physiotherapy treatment, with a consequent socio-economic impact. However, in order to carry out a clinical validation with a homogeneous sample, we target patients with severe knee osteoarthritis undergoing total knee replacement. This is justified, on the one hand, by the age of the population, which is proposed to be older adults. This will avoid unnecessary travel and will encourage compliance with recommendations such as social distancing. In addition, it satisfies the clinical need to perform physiotherapy after a highly invasive surgical procedure, which should preferably be done at home to optimize resources and reduce social and health risks.

## Methods

### Design

This design of this 2-armed randomized clinical trial has been approved by the Research Ethics Committees of Hospital Clínic i Universitari (no. 2020/350) and Hospital Politècnic i Universitari from Valencia (no. 2021-001-1), ensuring compliance with the ethical and legal provisions of the research. The experiments will be performed in accordance with the principles of the Declaration of Helsinki. The study has been prospectively registered (clinicaltrials.gov id. NCT05363137). Informed consent will be obtained from participants. The Universitat de València is responsible for the integrity and conduct of the study, which started on September 2022. The intervention period, in which participants will took part sequentially, will end approximately one year later, while the follow-up extends up to one year after enrolling the last participant.

### Participants

**Fig. 1:**
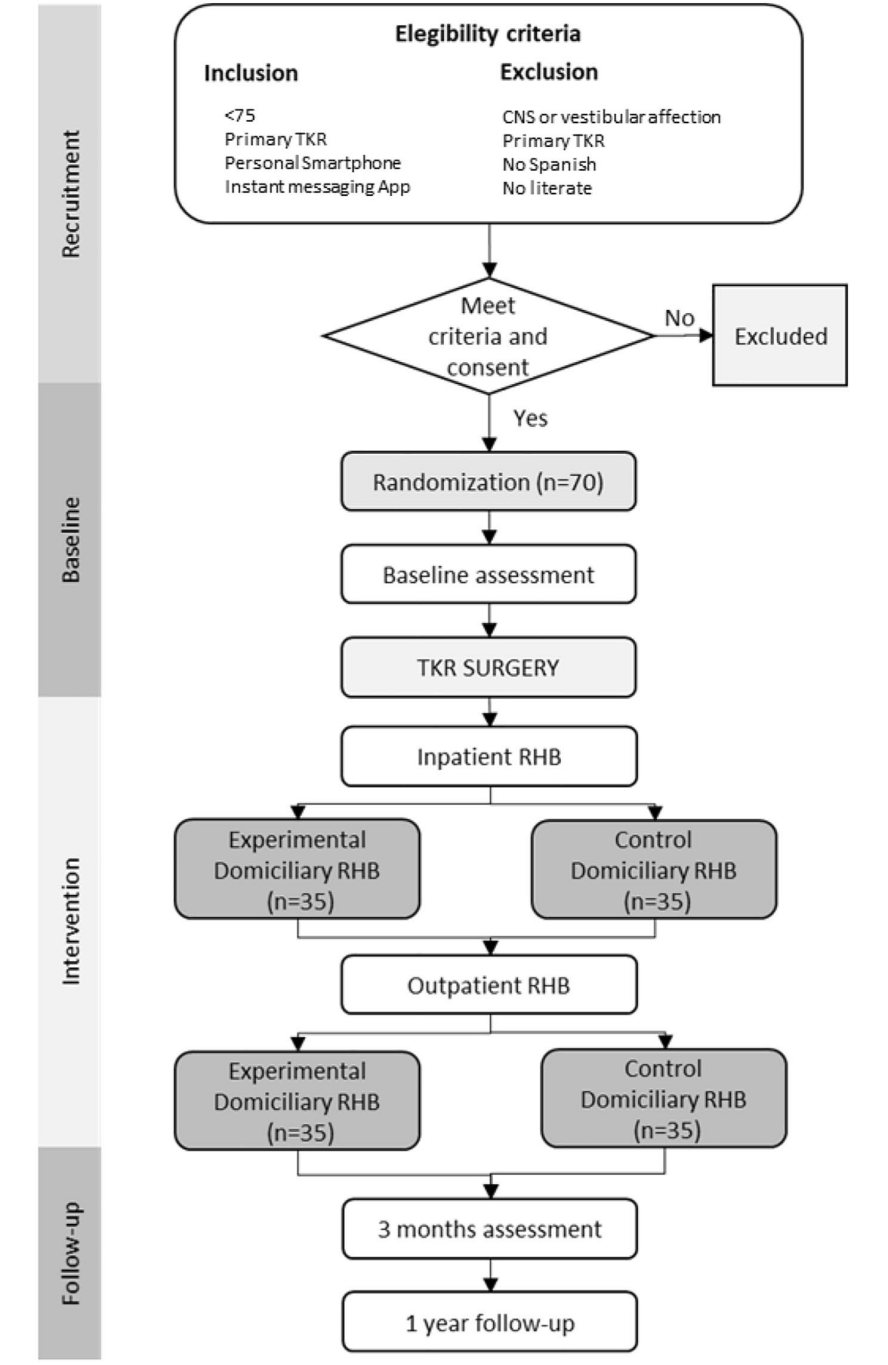
Flowchart of participants

Individuals on the waiting list for total knee replacement surgery will be invited if the following inclusion criteria are met: age less than 75, undergoing primary total knee replacement surgery, have a personal smartphone, with an instant messaging application installed, and familiar with the use of such application (more than 3 accesses per week), with no evident cognitive state that prevents to understand care provider instructions, read and write and able to consent to participate. Potential participants with vestibular or central nervous system affection (e.g., stroke), who cannot read or write, or do not understand Spanish, will be excluded. Figure 1 shows the participants flowchart.

### Procedures

Two orthopedic surgeons (one per hospital) will screen potential participants; then, the principal investigator will verify compliance with the inclusion criteria. informed consent will be obtained from the participants. A simple randomization method will be conducted, according to which a random number generator sequence is generated in origin with a software program (Matlab®). The principal investigator will allocate participants according to this sequence. One member of the team, blinded to group allocation, will have the only task to collect data and assess participants. Both groups will receive standard postoperative care, and the interventors will be blinded to group allocation. However, one member of the team, who will not be involved in standard care sessions, will show participants in the experimental group how to use the chatbot tool designed for this trial.

### Interventions

Both groups will receive standard postoperative care. Overall, standard in-patient rehabilitation begins the after surgery and lasts until medical discharge (usually less than 3 days). The patients are encouraged to train and taught how should keep training at home. Approximately one week after surgery, patients are appointed to start a standard outpatient rehabilitation program, in which mainly strengthening, functional and balance exercises are performed. After this, there is one more education session, in which the physiotherapist explains and instructs the exercises that they should continue doing at home, and facilitates a printed brochure with the information. The participants in the experimental group will receive the same education (i.e. last day before inpatient and outpatient discharge), but instead of being provided with a brochure, they will be informed that the intervention program will be supervised via an automated chatbot, through which they will receive motivational messages, reminders, and instruction on how to train. They will also be requested to answer some question (necessary to collect information such as compliance), as if it were a conversation. The tool allows participants to send messages with open questions or directly asking for help; if some message was not within the predefined possibilities, the physiotherapist receives a notification, and could contact via this messaging service or by telephone call as soon as possible.

### Data collection and measures

The basic characteristics of participants, including sex, age, weight and height (to calculate body mass index), previous falls and description (6 moths), operated knee, occupation and residence (rural/city) will be collected. The summary of measures and time points for assessment are shown in Table 1.

**Table 1:** Measures and assessment time points

	Enrolment	Baseline	3 m	12 m
Eligibility screen	x			
Informed consent	x			
Randomization	x			
Patient characteristics		x		
**Adherence-related measures**				
Compliance (Primary)			x	x
IPAQ-E		x	x	x
ACTi graph monitor			x	
**Evaluation of the tool**				
System Usability Scale			x	
Safety				x
Feasibility	x		x	x
**Clinical outcome**				
WOMAC		x	x	x
Timed up and go		x	x	
Five times sit-to-stand test		x	x	
Knee ROM		x	x	
Pain		x	x	x

*Abbreviations: IPAQ-E: International physical activity questionnaire; WOMAC: Western Ontario and McMaster Universities Osteoarthritis Index; ROM: Range of motion*

#### Adherence-related measures

The primary outcome will be the compliance with the intervention (i.e. the number of implemented sessions *n* and the rate between *n* and the programmed sessions). Adherence will be achieved for participants who comply over 80% of sessions goal, which is consistent with the definition of adherence from a pharmacological perspective [31, 32]. In the experimental group, compliance will be registered with the instant messaging tool. In the control group, a calendar will be provided to participants, who will be instructed to register every training day and time. Participants who document the number of days they train, with over 80% of documented sessions will be classified as achieved adherence.

The Spanish version of the International Physical Activity Questionnaire (IPAQ-E) will assess the types of intensity of physical activity and sitting time as part of the participants’ daily lives, i.e., the total physical activity in metabolic equivalent of task (MET)-minutes/week and time spent sitting. ActiGraph wGT3X-BT monitors will be provided to participants to capture and record physical activity parameters, since this is a reliable and valid measurement of PA in older adults after TKR [33].

In addition, we will assess the system usability, feasibility and safety. Safety will be assessed by reporting the number of adverse events recorded from baseline to 12 m, these being was defined as any unfavorable or unintended diagnosis, sign, symptom, or disease associated with the study. Feasibility will be evaluated with recruitment and retention rates. The system usability scale will be used for measuring the usability of the tool, since this is a reliable tool to evaluate a wide variety of products and services, including hardware, software, mobile devices, websites and applications; [34] it consists of a 10-item questionnaire with five response options from strongly agree to strongly disagree. The sores are added together and then multiplied by 2.5 to convert the original scores to 0-100. Based on research, a score above a 68 would be considered above average and anything below 68 is below average, however the results will be normalized as a percentile ranking. In addition, we will collect information on the number of interactions with the tool per patient, total accesses, the percentage of the originally specified treatment that has been completed, as well as the total protocols completed.

#### Clinical outcome

The following measures will be used to assess function and pain in order to determine the effects on clinical outcome. The Western Ontario and McMaster Universities Osteoarthritis Index (WOMAC), a self-administered questionnaire consisting of 24 items divided into pain, stiffness and physical function will register self-reported status [35]. The Timed up and go test, a timed test of general mobility in which the participant is instructed to get up from an arm-chair, walk three meters, turn around a cone, and come back to sit again [36]. The Five times sit to stand test will be used to assess functional performance, as a valid instrument that test lower limb strength and balance [37]. Knee range of motion will be the clinician-reported measure used to assess knee function with a digital goniometer (Baseline ®) [38]. Knee pain will be assessed with a visual analogue scale, which ranges from, no pain, to 10, the worst possible pain, felt in the knee during the last week. Timepoints for assessment are shown in Table 1.

### Data analysis

A descriptive analysis of the characteristics of the participants will be conducted using means, standard deviations, frequencies, interquartile ranges and contingency tables. To test the hypothesis that the participants in the experimental group will have a higher compliance and, consequently, better clinical outcome in terms of functionality and pain than participants in control group, an inferential analysis will compare baseline status with *t*-tests that will be parametric or not depending on the Wilcoxon and Shapiro-Wilk results. Subsequently, baseline progression will be compared with an analysis of variance (ANOVA) based on a general mixed model of repeated measures, looking for time (pre-post), group (experimental-control) and time per group interactions. In case of baseline differences, an analysis of covariance (ANCOVA) will be performed in which the data will be adjusted to the baseline scores, which will be entered as covariates. Post-hoc analysis will be performed in case of significant differences (Dunett and Tukey). Significance levels will be set at 95%. Quantitative adherence data will be compared in the same way (number of sessions, etc.). Additionally, a qualitative analysis will be used to estimate whether basal changes exceed the minimum detectable change or the minimum of clinical important difference. The questionnaires will be analyzed qualitatively.

To deal with missing data, in the event that a participant cannot, or decides not to attend the follow-up evaluation, we will try to get them to solve self-reported questionnaires at home. The possibility of displacement of the evaluator is also considered. In addition, the baseline characteristics of those patients who have been evaluated and those who have not will be compared, looking for possible sources of bias. A record of the reasons for dropping out will be made and, if necessary, the best analysis mechanism will be evaluated, with methods to impute data and re-analyze them with intention to treat, evaluating the possible impact on the conclusions. The incidence of clinical adverse events will be recorded and categorized as definite, probable, or possibly related to the interventions. In addition, the number and type of errors in the tool, failures and the number of times that there has been a need for reprogramming will be analyzed. The difficulties in use reported by the participants will be synthesized according to established scales.

If it is considered that the tool can increase the compliance ratio with the domiciliary physiotherapy program by 20%, a priori sample size calculation with the G*Power tool, using a F-test, repeated measures between groups, with *f =* 0.3, *α* = 0.05 and *β* = 0.2, considering an overall retention rate of 85%, and rounding up, suggests that an estimated of 70 participants equally distributed into the two groups will be needed to achieve a power over 80%. A post-hoc analysis will determine the actual trial power.

## Discussion

Adherence to treatment and compliance with rehabilitation continue to be one of the main barriers in the recovery of patients with musculoskeletal disorders [5]. Even more, taking into account the current limitation of resources, which makes it less and less feasible for public health services to offer face-to-face rehabilitation [11]. And yet, a considerable number of studies suggest that domiciliary rehabilitation programs can be as effective as outpatient, supervised or even group rehabilitation programs [39]. But success largely depends on compliance with exercise programs [7–9].

In recent years, special attention has been paid to how to solve the problem of adherence, with multiple and very diverse proposals in different areas of health and conditions, not only in MD, but also neurological patients or even in patients with behavioral disorders [30]. We believe that both the evaluation of new strategies and the use of ICT are factors that will be key to overcoming this barrier.

The use of a virtual assistant is not usual in healthcare, but it is true that it is not something new either. A number of research has appraised this technology in different forms for different conditions. However, its use via an instant messaging service to treat musculoskeletal conditions has not been comprehensively apprised. It is quite likely that in the coming years there will be an increase and generalization in the use of virtual assistants and conversational Chabots in healthcare. So it becomes necessary to generate scientific evidence on aspects such as usefulness, usability, validity and effectiveness in different areas.

### Strengths and limitations

The proposal is innovative; is positioned at the forefront of remote physiotherapy treatment, being aware that technological advances will be integrated progressively. In addition, it takes into account both the current resource limitation and possible health situations similar to the COVID-19 pandemic, which would require remote treatment. Therefore, through an initiative that provides a solution to current and future needs, this research responds to the requirements of an aging society. In addition, the proposal is evaluated in end users, in order to improve the capabilities of older adults with chronic MD in their daily lives. It is, therefore, a current and groundbreaking project, perfectly aligned with social and health priorities.

The interventions have been designed in a realistic way, taking into consideration the information provided by long-experienced physiotherapists, orthopedic surgeons and researchers, but also patient feedback in previous studies [40]. The experimental intervention has been designed as similar as possible to standard outpatient care, and adapted to home environment. Inclusion criteria are broad, not restricted to a large number of patients with comorbidities, which will increase the applicability. The exclusion criteria did not seek to promote the results, and therefore, there is no exclusion due to social disadvantages, depression, anxiety or poor motivation, which are frequently set in clinical research.

This research comes with limitations, for instance, the interventions cannot be concealed, and that some of the measures are usual in research but not clinical practice. We plan to share, critically appraise and disseminate the results by offering critical discussion of the findings, description of potential clinical impact, clinical application, and contextualization within contemporary literature [40]. A protocol publication helps to reduce implementation bias; however, in case of substantial changes in study design and protocol, these will be reported with reasons.

## Electronic supplementary material

Below is the link to the electronic supplementary material.

Supplementary Material 1

## Data Availability

Data sharing is not applicable to this article as no datasets were generated or analyzed during the current study. Any information can be requested / consulted to the principal investigator or corresponding author.

## List of abbreviations

MD: Musculoskeletal disorders
ICT: Information and communication technologies
NLP: Natural language processing
IPAQ-E: International Physical Activity Questionnaire
WOMAC: The Western Ontario and McMaster Universities Osteoarthritis Index
